# A 2-V 1.4-dB NF GaAs MMIC LNA for K-Band Applications

**DOI:** 10.3390/s23020867

**Published:** 2023-01-12

**Authors:** David Galante-Sempere, Sunil Lalchand Khemchandani, Javier del Pino

**Affiliations:** Institute for Applied Microelectronics (IUMA), Department of Electronics and Automatic Engineering, University of Las Palmas de Gran Canaria (ULPGC), Campus Universitario de Tafira, 35017 Las Palmas de Gran Canaria, Spain

**Keywords:** low noise amplifier, noise figure, monolithic microwave integrated circuit, gallium arsenide, electromagnetic simulation, input return loss, K-band, 5G

## Abstract

A 1.4-dB Noise Figure (NF) four-stage K-band Monolithic Microwave Integrated Circuit (MMIC) Low-Noise Amplifier (LNA) in UMS 100 nm GaAs pHEMT technology is presented. The proposed circuit is designed to cover the 5G New Release n258 frequency band (24.25–27.58 GHz). Momentum EM post-layout simulations reveal the circuit achieves a minimum NF of 1.3 dB, a maximum gain of 34 dB, |*S*_11_| better than –10 dB from 23 GHz to 29 GHz, a *P*_1dB_ of –18 dBm and an OIP3 of 24.5 dBm. The LNA draws a total current of 59.1 mA from a 2 V DC supply and results in a chip size of 3300 × 1800 µm^2^ including pads. We present a design methodology focused on the selection of the active device size and DC bias conditions to obtain the lowest NF when source degeneration is applied. The design procedure ensures a minimum NF design by selecting a device which facilitates a simple input matching network implementation and obtains a reasonable input return loss thanks to the application of source degeneration. With this approach the input matching network is implemented with a shunt stub and a transmission line, therefore minimizing the contribution to the NF achieved by the first stage. Comparisons with similar works demonstrate the developed circuit is very competitive with most of the state-of-the-art solutions.

## 1. Introduction

Although SOI technologies achieve remarkable results and are closing the gap with existing III-V processes [1], the latter still show a superior performance and are attracting the attention of many integrated circuit designers. Moreover, monolithic microwave integrated circuit (MMIC) processes provide an efficient solution for implementing discrete components and full radio interfaces in a single chip [2]. In this sense, gallium nitride (GaN)-based circuits provide many advantages, mainly in terms of power management, efficiency, and breakdown voltage [3]. In contrast, gallium arsenide (GaAs) technologies are the preferred option when a lower noise figure (NF) is pursued at greater frequencies [4,5]. Therefore, GaAs technologies are most appropriate in high-frequency scenarios requiring a very low NF, such as mmWave applications, SATCOMs or 5G networks. These technologies are utilized in high-resolution radar, including short range military aircraft radios and astronomical observations, all of them operating in the K-band. Particularly, the 26 GHz frequency band is of great interest since it has been identified as a pioneer band for the European Union’s 5G new radio (NR) networks [6]. There are two mm-Wave bands, designated as n258 and n257 in 3GPP NR, ranging from 24.25 to 27.5 GHz and 27.5 to 29.5 GHz, respectively. They enable very high data rates and data capacity, making them suitable for hotspot coverage. Similarly, the US identified the 27.5–28.35 GHz band for the same purpose, whereas the 27.5–29.5 GHz and 26.5–29.5 GHz frequency bands are considered for Japan and Korea, respectively [6].

To implement a very low noise receiver, special attention must be paid to the design of the low-noise amplifier (LNA) since its noise contribution is critical to the NF of the system. Thus, the LNA performance affects the overall receiver sensitivity and linearity [2,7,8]. Not many works are available in the literature that report LNA implementations achieving a NF below 1.5 dB with a gain above 30 dB and low Input Return Loss (IRL or |*S*_11_|) [4,5,9,10,11,12,13].

In this article, a 2-V 1.4-dB NF four-stage GaAs MMIC LNA from 24.25 to 27.5 GHz is presented. We introduce a design methodology focused on the selection of the active device geometry and DC bias conditions to obtain the lowest NF possible in a common-source (CS) amplifier with source degeneration. The LNA operates in the K-band (from 23 to 29 GHz), achieving a maximum gain of 34 dB at 24.5 GHz, a minimum NF of 1.3 dB at 26.5 GHz, an |*S*_11_| better than 10 dB, a *P*_1dB_ of –18 dBm and an OIP3 of 24.5 dBm. In Section 2, a detailed description of the proposed circuit is presented, and particular aspects regarding the design implementation with the selected Process Design Kit (PDK) are provided in Section 3. Electromagnetic (EM) post-layout simulation results of the four-stage GaAs LNA are shown in Section 4, and a comparison is made with the most relevant proposals available in the literature. Conclusions are drawn in Section 5.

## 2. Circuit Design

According to the Friis formula [2,7], the overall noise figure (*NF_tot_*) of a cascaded system with *n* stages is given as (1), where *NF_n_* and *G_n_* represent the NF and gain of the *n*-th stage, respectively. As seen, the first element contributes the most to *NF_tot_*, and the NF of the upcoming stages is attenuated by the gain of the first stage (*G*_1_) and all the preceding stages. Hence, the implementation of the LNA must be thoroughly carried out since its performance is critical for the receiver [2,7].

(1) $$NF_{tot}=1+\left(NF_{1}-1\right)+\frac{NF_{2}-1}{G_{1}}+\ldots +\frac{NF_{n}-1}{G_{1}\cdot \cdot \cdot G_{n-1}}$$

The LNA is composed of four cascaded CS stages with source degeneration to achieve a minimum NF design. The Friis formula can be applied to the whole system but it can be applied locally too, as shown in (2).

(2) $$NF_{LNA}=1+\left(NF_{s1}-1\right)+\frac{NF_{s2}-1}{G_{s1}}+\frac{NF_{s3}-1}{G_{s1}\cdot G_{s2}}+\frac{NF_{s4}-1}{G_{s1}\cdot G_{s2}\cdot G_{s3}}$$

If the LNA is divided into four CS amplifiers, the first stage (characterized by *NF_s_*_1_*, G_s_*_1_) is again the main contributor to the overall LNA NF (*NF_LNA_*). Applying (2), one can calculate the requirements of each stage to achieve a certain value of *NF_LNA_*. We assume the designer is interested in *NF_LNA_* = 1.4 dB and *G_tot_* = 33 dB. If the first stage presents *NF_s_*_1_ = 1 dB and *G_s_*_1_ = 6.5 dB (which is constrained by the selected process and the active device selection), assuming *NF_s_*_2_ = *NF_s_*_3_ = *NF_s_*_4_ = *NF_s_*_234_ and *G_s_*_2_ = *G_s_*_3_ = *G_s_*_4_ = *G_s_*_234_ for simplicity, then to achieve the desired performance, the *NF_s_*_234_ and *G_s_*_234_ should be:

(3) $$\{\begin{array}{c} NF_{s234}<1.7\;\left(dB\right)\\ G_{s234}\geq 9\;\left(dB\right) \end{array},$$

In this sense, the first stage should introduce the lowest NF possible and a high gain in order to allow a more flexible design of the upcoming stages. Additionally, to achieve *NF_s_*_1_ = 1 dB and *G_s_*_1_ = 6.5 dB, the implementation of the input matching network with a single inductor is desired to minimize the number of elements in the signal path and to minimize the noise contribution of the input matching network. Otherwise, obtaining an LNA with an NF under 1.4 dB is not possible without lowering the NF of stages 2, 3 and 4. However, as explained in the following section, lowering *NF_s_*_234_ is only possible at the expense of a gain reduction, which means an additional stage may be needed to reach *G_tot_* = 33 dB.

The performance of the active device used in the CS amplifier is mainly defined by three parameters: the minimum NF it provides (*NF_min_*), the maximum stable gain (*G_max_*) and Rollet’s stability factor (*k*). The value of these three parameters depends on several factors, namely, the device geometry, physical parameters of the selected process, impedance matching or DC bias conditions, among others. The value of *NF_min_* is given as (4), where *g_m_* is the device transconductance (obtained from the device’s DC operating point), *R_G_* and *R_S_* are the gate and source resistances, *f* is the frequency of operation and *f_T_* is the transit frequency [1]. Similarly, *G_max_* is defined as (5), and Rollet’s stability factor *k* is given by (6).

(4) $$NF_{min}=1+K\sqrt{g_{m}\cdot \left(R_{G}+R_{S}\right)}\cdot \frac{f}{f_{t}}$$

(5) $$G_{max}=\left|\frac{S_{21}}{S_{12}}\right|\cdot \left(k-\sqrt{k^{2}-1}\right)$$

(6) $$k=\frac{1-{\left|S_{11}\right|}^{2}-{\left|S_{22}\right|}^{2}+{\left|S_{11}S_{22}-S_{12}S_{21}\right|}^{2}}{2\left|S_{21}S_{12}\right|}>1$$

Impedance matching selection plays a critical role in the design of each stage of the LNA. To obtain an amplifier that yields the maximum gain, one must match the input of the LNA to (*S*_11_)*, whereas to obtain a minimum NF design, one must match the input to the conjugate of the optimum source impedance (*S_opt_*)*. Nevertheless, these two values (*S*_11_ and *S_opt_*) are generally different, so that simultaneously matching the circuit for *NF_min_* and *G_max_* is not possible. Therefore, source degeneration is often applied to the CS amplifier to bring these two impedances close together. To be able to use a single inductance to implement the input matching network, the selected source impedance should present a real part close to 50 Ω so that a single gate inductor is enough to cancel the capacitive component [1]. The main problem is that most designs use fixed transistor sizes, so their *S_opt_* may not present a real part close to 50 Ω, resulting in a non-minimum NF design. To address this issue, multiple iterations of the design process are performed to find a combination of device size and input matching network that meets the appropriate NF specifications.

### Design Approach

**Figure 1:**
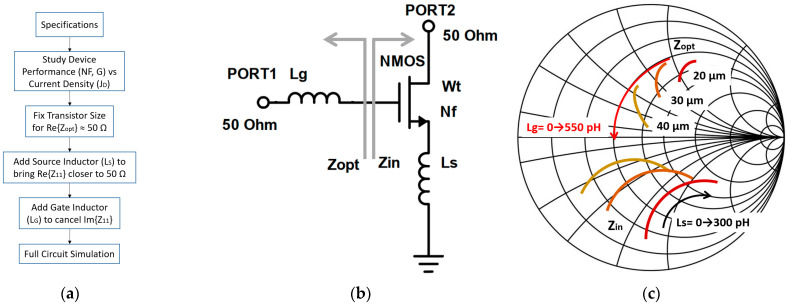
Simplified overview of the design procedure (**a**), schematic diagram used for device selection (**b**) and Smith Chart representation of the influence of varying the transistor width (*W_t_*), the source inductor (*L_s_*) and the gate inductor (*L_g_*) on the input impedance (*Z_in_*) and the optimum source impedance (*Z_opt_*) (**c**).

In this paper, we present a design methodology focused on how to choose the active device geometry and DC bias conditions to obtain the lowest NF possible when source degeneration is applied. A simplified overview of the design procedure is given in Figure 1a. First, to select the device size, the CS amplifier with the source degeneration depicted in Figure 1b is employed. Note that all the ports are matched to 50 Ω and the DC biasing circuitry is not shown for simplicity. The device geometry is defined by the total width (*W_t_*), which is the product of the unitary finger width (*W_u_*) and the number of fingers (*N_f_*). *Z_opt_* is the impedance seen looking away from the gate and *Z_in_* is the impedance seen looking into the gate of the active device. The effect of increasing *W_t_*, the value of the source inductor (*L_s_*) and the gate inductor (*L_g_*) are shown in Figure 1c.

**Figure 2:**
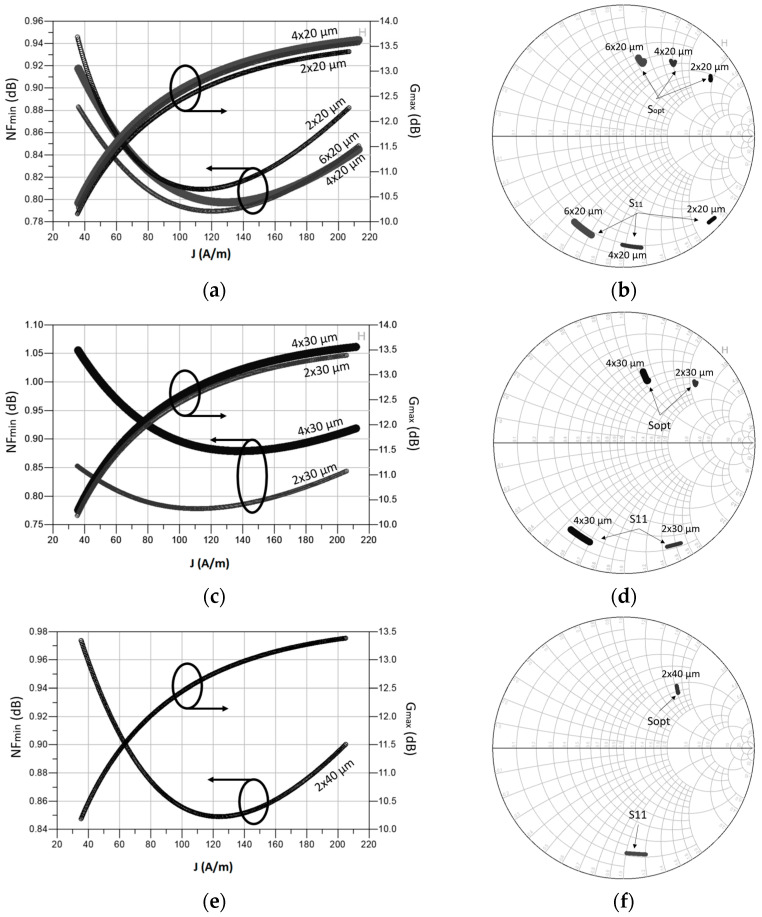
Current density and *S*_11_, *S_opt_* of the first stage transistor for a finger width of 20 µm (**a**,**b**), 30 µm (**c**,**d**) and 40 µm (**e**,**f**).

In Figure 1b, the source degeneration inductance *L_s_* is swept from 0 to 300 pH. Source degeneration helps reduce the NF at the expense of a gain reduction, improving the amplifier’s stability. A value of approximately 150 pH for *L_s_* is enough to bring the *S*_11_ and *S_opt_* closer to the unity circle, i.e., to achieve a real part of about 50 Ω. A gate inductor is then used to bring both *S*_11_ and *S_opt_* to the center of the Smith chart, obtaining a maximum gain and minimum NF amplifier. To select the size and biasing of the LNA first stage device, its performance (*NF_min_* and *G_max_*) against its current density (*J*) is studied, keeping close attention to the *S*_11_ and *S_opt_*, as shown in Figure 2, for different device sizes. In these figures, only device geometries that provide *S*_11_ and *S_opt_* close to 50 Ω are included.

A finger width of 20 µm is explored in Figure 2a,b. In Figure 2a, the *NF_min_* and *G_max_* are plotted as a function of the current density. As seen, a device of 4 fingers and 20 µm biased with a current density of 0.12 mA/µm results in a *G_max_* of 12.7 dB and 0.79 dB of *NF_min_*, which is the lowest *NF_min_* possible. As shown in Figure 2b, a device of 2 fingers × 20 µm would require a very high value of *L_g_* to be matched. It presents a real part higher than 50 Ω and would be increased further after applying source degeneration, so this choice is discarded. However, 6 and 4 finger devices present a real part lower than 50 Ω and would be suitable for the first stage design. In fact, both options could be matched to the input with a single gate inductor with a very similar value. Nevertheless, since the 4 × 20 µm transistor presents a *S_opt_* closer to the unitary circle, a lower value of *L_s_* can be employed. This situation is highly desirable since a very high *L_s_* decreases the NF, but it also decreases the gain significantly. In Figure 2c,d, a finger width of 30 µm is explored. Note that a 2 × 30 µm transistor results in an even lower *NF_min_* than the 4 × 20 µm device. Still, this device presents a real part higher than 50 Ω, so it is discarded for the same reason as the 2 × 20 µm transistor. The 4 × 30 µm transistor would be a better option, but its *NF_min_* is higher than the 4 × 20 µm device. Finally, a 2 × 40 µm transistor is studied in Figure 2e,f. Further geometries are discarded directly since their Re{*S_opt_*} are significantly lower than 50 Ω, making them unsuitable for the design. In addition, a higher total width yields a higher power consumption for the same current density.

From this analysis the designer can determine the optimal device and current density for minimum NF, maximum gain, or a reasonable trade-off between NF, gain and power consumption. Note that, as previously discussed, a 4 × 20 µm transistor with a current density of 0.12 mA/µm (120 A/m) is required for minimum NF, which results in a *G_max_* of about 12.8 dB and *NF_min_* of ~0.8 dB. However, after applying source degeneration, the value of *G_max_* is greatly reduced. A drain current (*I_D_*) of 4 (fingers) × 20 (µm) × 0.12 (mA/µm) = 9.6 (mA) is needed to bias the active device. In this step, it is critical to fix a device size and current density which facilitate the input matching considering the discussion of Figure 1b [1]. Once the components of the first stage are selected, the same procedure is followed to determine the device size and biasing of the upcoming stages to define their optimum source and load impedances to meet (3). When they are known, the interstage matching network can be designed. The last step is the design of the DC bias lines to feed the devices of each stage. In our circuit, quarter-wavelength lines with bypass capacitors were used to bias the gate and drain of each transistor. The transmission lines (TLs) used in the bias paths show high impedance in the LNA’s operating frequency band, and therefore, they do not contribute to impedance matching significantly.

## 3. Proposed Circuit

**Figure 3:**
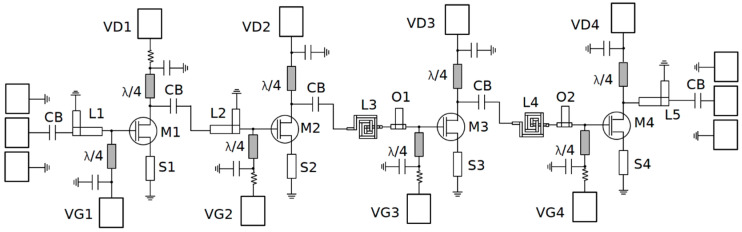
Proposed four-stage GaAs LNA detailed schematic.

**Figure 4:**
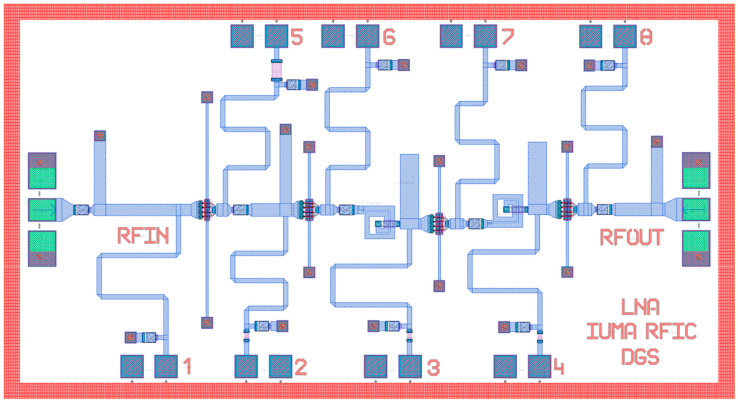
Layout of the Low-Noise Amplifier. Pads 1, 2, 3 and 4 represent the terminals VG1, VG2, VG3, and VG4, respectively. Also, pads 5, 6, 7 and 8 represent the terminals VD1, VD2, VD3, and VD4, respectively.

The amplifier is implemented using the models of the 100-nm UMS PH10 GaAs pHEMT process, characterized by a 130-GHz transit frequency (*f_T_*) and 1-dB NF @ 30 GHz. The schematic implementation of the proposed circuit is depicted in Figure 3, and the corresponding layout is shown in Figure 4.

Ground-signal-ground (GSG) pads with 150-µm pitch are used for the input and output RF signals. The gate voltages of each stage are provided through DC pads VG1, VG2, VG3, and VG4. Similarly, DC pads VD1, VD2, VD3 and VD4 are used to bias the drain voltage of each stage with 2 V. Instead of relying on inductors to bias the active devices as RF chokes, we use quarter-wavelength (λ/4) TLs with a length of 1 mm and a shunt capacitor of 0.53 pF. These bias lines are oversized to comply with the maximum ratings defined by the process design kit (PDK) to avoid electro-migration and aging issues. The main advantage of using λ/4 TLs is that, if designed correctly, they barely affect the matching networks and result in an almost negligible impact on the LNA’s overall NF and gain. Additionally, in order to provide DC isolation, a 0.58-pF DC block capacitor (CB) is used in the interstage matching networks as an additional component.

Since a very low NF is pursued, the designer must carefully choose the bias currents and inductances for each stage. Therefore, the first stage employs a device of four fingers with 5 µm each, resulting in a total width of 20 µm, and is biased with a current density of 0.47 mA/µm for minimum NF and low-power operation. Since the first stage achieves a very low NF (about 1 dB @ 26 GHz) but a limited gain (about 6.5 dB for the same frequency), the next stages are biased with a higher current density of 0.53 mA/µm to obtain a 9-dB gain-per-stage at the expense of a slightly higher NF. The device geometry selected for stages 2, 3 and 4 is 4 × 7.5 µm. Note that a *V_G_* and a *V_D_* of 0 V and 2 V are used to bias to all the transistors, respectively. Source degeneration is applied to all the CS stages since it prevents instability, reduces the NF, brings the *S*_11_ and *S_opt_* closer together and increases the circuit resilience to process, voltage, and temperature (PVT) variations. The source transmission line (S1 in Figure 3) adds a small inductance which brings the *S*_11_ of the input transistor closer to *S_opt_*_,_ as discussed in Figure 2b. The size of S1 is 420 × 10 µm^2^, which is enough to ensure stability and a low NF for the first stage. The transmission lines S2, S3 and S4 are sized 200 × 10 µm^2^, providing a lower inductance for a higher gain than the first stage. The selected source impedance for the first stage is equivalent to the device’s *S_opt_* and is given by (7). As seen, thanks to careful device sizing plus source degeneration, the real part of *Z_s_*_1_ is very close to 50 Ω. From the selected source impedance, one can obtain the source reflection coefficient ($\Gamma_{S}$) and then the corresponding load reflection coefficient ($\Gamma_{L}$) by applying expression (8). After translating $\Gamma_{L}$ into the load impedance *Z_L_*_1_, Equation (9) is obtained. Following this methodology, the source and load impedances for stages 2, 3 and 4 are obtained as (10) and (11), respectively. Since the interstage matching does not require any intermediate 50 Ω termination, the stages are directly matched to each other.

(7) $$Z_{S1}={\left(Z_{in}\right)}^{*}=50\cdot \left(1.043+j\cdot 1.399\right)\;(Ω)$$

(8) $$\Gamma_{L}={\left(S_{22}+\frac{S_{12}S_{21}\Gamma_{S}}{1-S_{11}\Gamma_{S}}\right)}^{*}$$

(9) $$Z_{L1}={\left(Z_{out}\right)}^{*}=50\cdot \left(1.366+j\cdot 1.261\right)\;(Ω)$$

(10) $$Z_{S2,3,4}=50\cdot \left(0.329+j\cdot 0.709\right)\;(Ω)$$

(11) $$Z_{L2,3,4}=50\cdot \left(0.537+j\cdot 0.955\right)\;(Ω)$$

**Figure 5:**
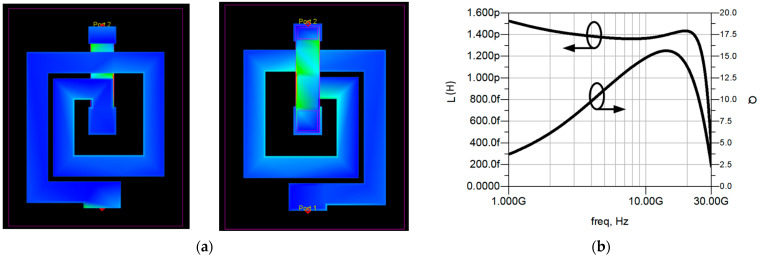
Layout and current distribution (**a**), and inductance, and quality factor (**b**) for the inductors employed in stages 3 and 4.

The input matching network can be implemented by a very low-inductance, high-Q inductor since it is critical that it presents a very high Q to avoid a significant degradation of the NF [14,15]. However, in practice, the inductance required is so small that it can be easily implemented with a series TL and a shunt stub. The same applies to the inductor between stages 1 and 2, which has been replaced with a series TL and a shunt stub. The matching networks between stages 2 and 3 and 3 and 4 were implemented using two inductors (520 pH and 510 pH, Q ≈ 15) and two open stubs (O1, O2) in series. The layout of these inductors, the current density distribution and the inductance and Q values obtained from the EM simulations are shown in Figure 5. The open stubs O1 and O2 are sized 80 × 280 µm^2^ and 80 × 270 µm^2^, respectively, and they provide an extra capacitance required for proper matching. Finally, a series TL, a shunt stub, and a DC Block capacitor of 0.58 pF form the output matching network. The layout of the circuit is depicted in Figure 4, with a total size of 3300 × 1800 µm^2^ including pads.

## 4. Simulation Results

**Figure 6:**
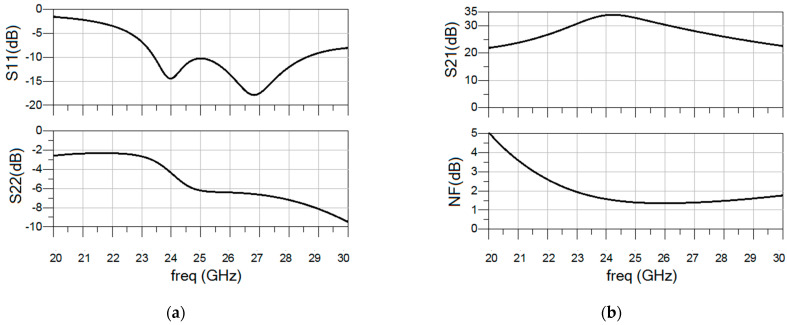
Input and output return loss (**a**), and gain and NF (**b**) of the proposed LNA from 20 to 30 GHz.

Post-layout scattering (S-parameters) and noise parameters were simulated using Keysight’s Advanced Design System software and the Momentum EM simulator at room temperature. The resulting |*S*_11_| and |*S*_22_| as well as the gain and NF are depicted in Figure 6a,b. The proposed LNA presents an IRL better than 10 dB from 23.5 to 28.5 GHz and an output return loss better than 5 dB. A maximum gain of 34 dB is obtained at 24 GHz, and an NF below 1.4 dB is obtained from 24 GHz to 28 GHz with a minimum value of 1.3 dB at 26.5 GHz.

**Figure 7:**
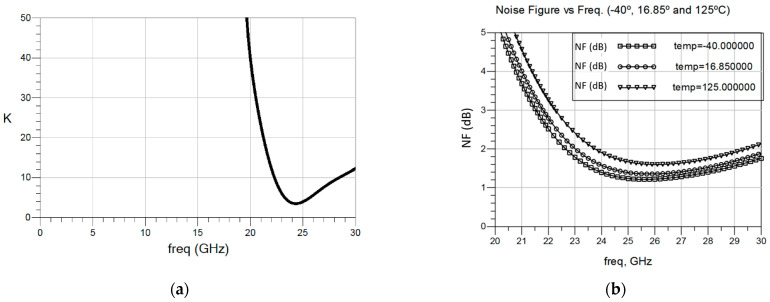
Stability factor (**a**), and NF for –40 °C, 16.85 °C and 125 °C (**b**) of the proposed GaAs LNA.

The value of the Rollet’s stability factor *k* is shown in Figure 7a. As seen, the *k*-factor is above one from 0 to 30 GHz, with a minimum of 3.5 at a frequency of 24.3 GHz. Individual stability was also checked for each stage and for the whole four-stage amplifier, concluding that the LNA is unconditionally stable. The NF of the LNA was simulated for different temperature values (–40 °C, 16.85 °C and 125 °C), as shown in Figure 7b. As expected, the best results are observed at the lowest temperature, with a minimum NF as good as 1.2 dB at 25.5 GHz. On the other hand, at 125 °C the LNA presents a minimum NF of 1.6 dB at 26 GHz.

**Figure 8:**
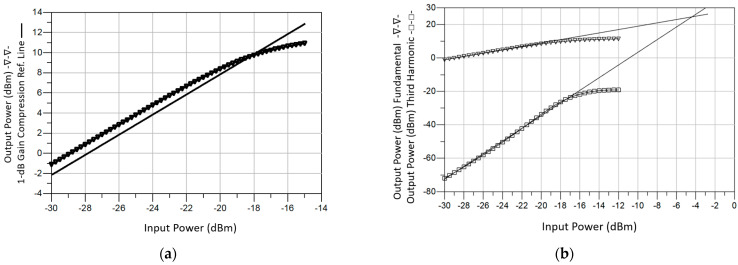
Output 1-dB compression point (*P*_1dB_) (**a**), and output third order intercept (OIP3) (**b**) of the proposed GaAs LNA.

The results of the single- and two-tone non-linear simulations for the proposed 4-stage GaAs LNA are shown in Figure 8. The gain compression of the LNA at 26 GHz is presented in Figure 8a, and the input and output third order intercept points (IIP3 and OIP3) are shown in Figure 8b, where two tones with 200 MHz spacing from a central frequency of 26 GHz were used. The GaAs LNA achieves an output power 1 dB-compression point (*P*_1dB_) of –18 dBm, an IIP3 of –4.5 dBm and an OIP3 of 24.5 dBm.

**Figure 9:**
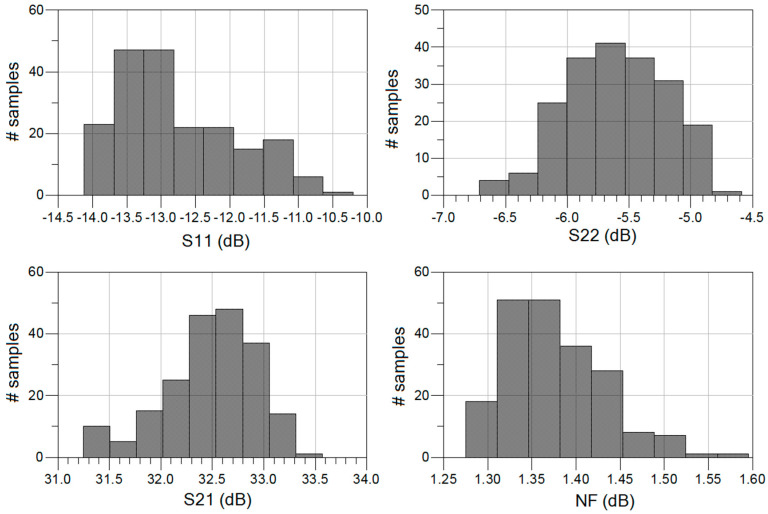
Monte Carlo results for 250 samples of the proposed 4-stage GaAs LNA.

A Monte Carlo analysis with 250 samples was also carried out to verify the results vary within an acceptable range. The histograms representing the input return loss, output return loss, gain and NF of the circuit are shown in Figure 9. As seen, the input return loss is better than 10 dB for all the samples, the output return loss is better than 5 dB for most of the samples, the gain is between 32 and 33 dB, and the NF is better than 1.4 dB for most of the samples.

To better understand how the proposed circuit performs in contrast to other works available in the literature, the small-signal figure of merit (*FoM_SS_*) defined in (12) is introduced [5]. A concise comparison with similar works available in the literature along with the main results of the proposed LNA are given in Table 1. The results of the proposed circuit are superior to most of the GaAs LNAs reported in the literature and are superior to the SOI LNA proposed in [16]. As seen in Table 1, although the 4-stage LNA presents a power consumption and area in line with most of the proposals available in the literature, it achieves one of the highest gains and lowest NFs reported, this combination results in the best *FoM_SS_* compared with the other proposals.

(12) $$FoM_{SS}=\frac{G}{NF-1}$$

## 5. Conclusions

This paper presents a four-stage LNA using a 100 nm GaAs pHEMT process that covers the 5G New Release n258 frequency band (24.25–27.58 GHz). The proposed LNA achieves a maximum gain of 34 dB, a minimum NF as low as 1.3 dB, an input return loss better than –10 dB from 23 to 29 GHz, a *P*_1dB_ of –18 dBm, and an OIP3 of 24.5 dBm. The LNA draws a total current od 59.1 mA from a 2V DC supply, resulting in a chip size of 3300 × 1800 µm^2^ including pads. Electromagnetic simulations as well as a Monte Carlo analysis results at room temperature demonstrate a final gain of 33 dB, a 1.4 dB NF, an |*S*_11_| better than 10 dB, an |*S*_22_| better than 5 dB, a *P*_1dB_ of –18 dBm and an OIP3 of 24.5 dBm in the band of interest. The paper also presents a design methodology focused on the selection of the active device size and DC bias conditions to obtain the lowest NF when source degeneration is applied. The design procedure ensures a minimum NF design by selecting a device that facilitates a simple input matching network implementation and obtains a reasonable input return loss thanks to the application of source degeneration. This approach minimizes the number of elements in the input matching network. Comparisons with similar works demonstrate the developed circuit is competitive with state-of-the-art solutions.

## Figures and Tables

**Table 1:** Comparison of similar works available in literature.

Parameter	[17]	[18]	[16]	[19]	[4]	This Work
Data	Simulated	Measured	Measured	Measured	Measured	Simulated
Freq. range (GHz)	26–36	15–25	23–30	17.5–22.5	24–30	23–29
Gain (dB)	33	30	12.8	23.9	25	33
Noise figure (dB)	1.8	1.5	1.5	1.3	1.5	1.4
|*S*_11_| (dB)	12	10	6	12	10	10
|*S*_22_| (dB)	12	10	6	5	10	5
Power consumption (mW)	-	212	15	66	150	118.2
Publication year	2015	2017	2018	2020	2022	2023
FoM_SS_	41.25	60	25.6	79.67	50	82.5
Process	GaAs 100 nm	GaAs 150 nm	SOI 45 nm	GaAs 90 nm	GaAs 70 nm	GaAs 100 nm
Area (mm^2^)	3.64	1.87	0.3021	2.6	-	5.94
